# Supplementary material for: Impact of blood glucose abnormalities on outcomes and disease severity in patients with severe sepsis: An analysis from a multicenter, prospective survey of severe sepsis
Source: PLoS One. 2020 Mar 11;15(3):e0229919. doi: 10.1371/journal.pone.0229919 (PMC7065801; doi:10.1371/journal.pone.0229919)
Supplement: S1 Table — (DOCX) [file pone.0229919.s001.docx]

Supplementary Table 1. Characteristics and blood glucose levels (mg/dL) on admission among patients with severe sepsis in 59 intensive care units in Japan (n= 1158)

| Characteristics | All patients  ( n= 1158 ) | <70 mg/dL  ( n= 69) | 70–139 mg/dL  ( n= 543 ) | 140–179 mg/dL  ( n= 233 ) | >180 mg/dL  ( n= 313) | P value |
| --- | --- | --- | --- | --- | --- | --- |
| Age | 73 (64-81) | 73 (66-79.5) | 72 (62-82) | 74 (64-81.5) | 74 (64.5-81) | 0.292 |
| Male sex (n, %) | 696, 60.1% | 34, 49.3%**,^#^ | 313, 57.6% | 149, 63.9% | 200, 63.9% | 0.045 |
| BMI | 21.8  (19.0-24.7) | 21.3  (19.1-24.8) | 21.3  (18.8-24.2) | 21.9  (19.5-25.1) | 22.4  (19.1-25.4) | 0.076 |
| Pre-existing diabetes mellitus (n, %) | 270, 23.3% | 13, 18.6%^#^ | 69, 12.7%^#^ | 45, 19.3%^#^ | 143, 45.7% | <0.001 |
| Admission source (n, %) |  |  |  |  |  | 0.094 |
| ED | 664, 57.3% | 45, 65.2% | 284, 52.3% | 141, 60.5% | 194, 62.0% |  |
| Transfer or other departments | 444, 38.3% | 23, 33.3% | 232, 42.7% | 80, 34.3% | 109, 34.8% |  |
| ICU | 48, 4.1% | 1, 0.4% | 25, 4.6% | 12, 5.2% | 10, 3.2% |  |
| Coexisting conditions (n, %) |  |  |  |  |  |  |
| Myocardial infarction | 57, 4.9% | 1, 1.4% | 26, 4.8% | 10, 4.3% | 20, 6.4% | 0.329 |
| Congestive heart failure | 124, 10.7% | 5, 7.2% | 52, 9.6% | 36, 15.5% | 31, 9.9% | 0.063 |
| Peripheral vascular disease | 29, 2.5% | 1, 1.4% | 16, 2.9% | 7, 3.0% | 5, 1.6% | 0.564 |
| Cerebrovascular disease | 136, 11.7% | 5, 4.2% | 42, 13.3% | 26, 11.2% | 33, 10.5% | 0.382 |
| Dementia | 97, 8.4% | 2, 2.9% | 54, 9.9% | 13, 5.6% | 28, 8.9% | 0.074 |
| COPD | 81, 7.0% | 4, 5.8% | 38, 7.0% | 19, 8.2% | 20, 6.4% | 0.847 |
| Connective tissue disease | 83, 7.2% | 6, 8.7% | 48, 8.8% | 13, 5.6% | 16, 5.1% | 0.145 |
| Peptic ulcer disease | 32, 2.8% | 1, 1.4% | 15, 2.8% | 5, 2.1% | 11, 3.5% | 0.698 |
| Diabetes mellitus without organ damage | 196, 16.9% | 6, 8.7%**^,#^ | 46, 8.5%**^,#^ | 37, 15.9% | 101, 34.2% | <0.001 |
| Diabetes mellitus with organ damage | 74, 6.4% | 7, 10.1%*^,^** | 23, 4.2%^#^ | 8, 3.4%^#^ | 36, 11.5% | <0.001 |
| Chronic kidney disease | 83, 7.2% | 4, 5.8% | 40, 7.4% | 20, 8.6% | 19, 6.1% | 0.683 |
| Hemiplegia | 43, 3.7% | 2, 2.9% | 22, 4.1% | 10, 4.3% | 9, 2.9% | 0.769 |
| Malignancy (solid) | 159, 13.7% | 8, 11.5% | 69, 12.7% | 42, 18.0% | 40, 12.8% | 0.202 |
| Malignancy (blood) | 8, 0.7% | 1, 1.4% | 2, 0.4% | 4, 1.7% | 1, 0.3% | 0.132 |
| Metastatic tumor | 25, 2.2% | 1, 1.4% | 9, 1.7% | 10, 4.3% | 5, 1.6% | 0.098 |
| Mild liver disease | 43, 3.7% | 2, 2.9% | 19, 3.5% | 13, 5.6% | 9, 2.9% | 0.379 |
| Moderate to severe liver disease | 26, 2.2% | 8, 11.6%*^,^**^,#^ | 9, 1.7% | 2, 0.9% | 7, 2.2% | <0.001 |
| AIDS | 1, 0.1% | 0, 0.0% | 1, 0.2% | 0, 0.0% | 0, 0.0% | 0.769 |
| CCI | 1(0-2) | 1(0-2) | 1(0-2) | 1(0-2) | 1(0-3) | 0.293 |
| ADL：Inactive (n, %) | 282, 24.4% | 10, 14.5% | 139, 25.6% | 60, 25.8% | 73, 23.3% | 0.003 |
| Suspected site of infection | (n, %) |  |  |  |  | 0.001 |
| Lung | 359, 31.0% | 20, 29.0% | 140, 25.8% | 82, 35.2% | 117, 37.4% |  |
| Abdomen | 303, 26.2% | 17, 24.6% | 159, 29.3% | 62, 26.6% | 65, 20.8% |  |
| Urinary tract | 216, 18.7% | 15, 23.2% | 107, 19.7% | 43, 18.5% | 50, 16.0% |  |
| Soft tissue | 115, 9.9% | 5, 7.2% | 66, 12.2% | 14, 6.0% | 30, 9.6% |  |
| Central nerve | 23, 2.0% | 0, 0.0% | 4, 0.7% | 3, 1.3% | 16, 5.1% |  |
| IV catheter | 19, 1.6% | 0, 0.0% | 10, 1.8% | 3, 1.3% | 6, 1.9% |  |
| Osteoarticular | 21, 1.8% | 2, 2.9% | 7, 1.3% | 6, 2.6% | 6, 1.9% |  |
| Endocardium | 15, 1.3% | 1, 1.9% | 7, 1.3% | 4, 1.7% | 3, 1.0% |  |
| Wound | 11, 0.9% | 0, 0.0% | 7, 1.3% | 2, 0.9% | 2, 0.6% |  |
| Implant device | 8, 0.7% | 0, 0.0% | 5, 0.9% | 3, 1.3% | 0, 0.0% |  |
| Other | 68, 5.9% | 8, 11.6% | 31, 5.7% | 11, 4.7% | 18, 5.8% |  |
| Positivity of blood cultures (n, %) | 681, 58.8% | 43, 62.3% | 338, 62.2% | 117, 50.2% | 183, 58.5% | 0.072 |
| Septic shock (n, %) | 727, 62.8% | 57, 82.6%*^,^**^,#^ | 351, 64.6% | 132, 56.7% | 187, 59.7% | 0.001 |
| Organ dysfunction on arrival (n, %) |  |  |  |  |  |  |
| Hypotension | 591, 51.0% | 47, 68.1%**^,#^ | 291, 53.6% | 111, 47.6% | 142, 45.4% | 0.002 |
| Decreased BP>40 mmHg | 210, 18.1% | 12, 17.4% | 106, 19.5% | 43, 18.5% | 49, 15.7% | 0.584 |
| Hyperlactatemia  (>2 mmol/L) | 784, 67.7% | 58, 84.1%*^,^**^,^ | 337, 62.1%^#^ | 162, 69.5% | 227, 72.5% | <0.001 |
| Acute kidney injury (Creatinine>2 mg/dL) | 444, 38.3% | 43, 62.3%*^,^**^,#^ | 195, 35.9% | 83, 35.6% | 123, 39.3% | <0.001 |
| Acute lung injury | 439, 37.5% | 23, 33.3% | 191, 35.2% | 96, 41.2% | 129, 41.2% | 0.044 |
| Hyperbilirubinemia  (>2.0 mg/dL) | 199, 17.2% | 23, 33.3%*^,^**^,#^ | 88, 16.2% | 46, 19.7% | 42, 13.4% | 0.001 |
| Thrombocytopenia (<100,000/μL) | 341, 29.4% | 33, 47.8%*^,^**^,#^ | 162, 29.8% | 68, 29.2% | 78, 24.9% | 0.002 |
| Coagulopathy (INR>1.5) | 217, 18.7% | 29, 42.0%*^,^**^,#^ | 116, 21.4% | 32, 13.7% | 40 12.8% | <0.001 |
| Lactate level (mmol/L) | 3.00  (1.80-5.30) | 5.50  (3.05- 9.75) | 2.70  (1.60- 5.10) | 2.80  (1.78- 4.93) | 3.25  (2.18-5.60) | <0.001 |
| qSOFA score | 2 (1-2) | 2 (1-2) | 2 (1-2) | 2 (1-2) | 1 (1-2) | 0.003 |
| APACHE II score | 23 (17-29) | 29(21.75-36) | 22 (16-29) | 22 (15-28) | 23(17-30) | 0.013 |
| SIRS score | 3 (2-4) | 3 (2-3.75) | 3 (2-3.5) | 3 (2-4) | 3 (3-4) | 0.032 |
| SOFA score | 9 (6-11.25) | 11.5 (9-14) * | 8(6-11) | 8(5-11) | 9 (6-11) | <0.001 |
| Compliance with all applicable elements of sepsis 3-h bundle | | | | (n, %) |  |  |
| Entire 3-h resuscitation bundle† | 535, 64.5% | 47, 75.8% | 240, 62.5% | 109, 69.9% | 139, 61.2% | 0.067 |
| B1. Serum lactate obtained | 1125, 97.2% | 68, 98.6% | 529, 97.4% | 222, 95.3% | 306, 97.8% | 0.261 |
| B2. Broad-spectrum antibiotic given | 970, 83.8% | 59, 85.5% | 452, 83.2% | 192, 82.4% | 267, 85.6% | 0.721 |
| B3. Blood cultures obtained before broad-spectrum antibiotic administration | 1064, 92.0% | 66, 95.7% | 498, 91.9% | 213, 91.4% | 287, 92.0% | 0.712 |
| B4. 30 mg/kg crystalloid fluid bolus delivered (yes/cases with indication)† | 636, 76.6% | 51, 82.3% | 295, 76.6% | 126, 80.8% | 164, 72.3% | 0.170 |
| Entire 3-h resuscitation bundle + vasopressors use + re-measured lactate | 392, 57.5% | 40, 75.5% | 188, 57.9% | 69, 60.0% | 95, 50.3% | 0.010 |
| Entire 3-h resuscitation bundle + vasopressors use | 444, 60.1% | 43, 75.4% | 211, 60.1% | 81, 61.8% | 109, 54.5% | 0.039 |
| Number of patients with corticosteroid requirement within 6 h (n, %) | 191, 16.5% | 30, 43.5%*^,^**^,#^ | 90, 16.6% | 24, 10.3% | 47, 15.0% | <0.001 |

Reported counts (proportions) for categorical and median (interquartile range) for continuous variables. †septic shock or lactate >4 mmol/L

Missing data: BMI=20, Admission source=2, ADL=1, blood culture=6, qSOFA=24, APACHE II=124, SIRS=0, SOFA=153

ICU=intensive care unit, BMI=body mass index, ED=emergency department, CCI=Charlson Comorbidity Index, COPD=chronic obstructive pulmonary disease; AIDS=acquired immune deficiency syndrome, ADL=activities of daily living, IV=intravenous, qSOFA=quick sepsis organ failure assessment, APACHE=acute physiology and chronic health evaluation, SIRS=systemic inflammatory response syndrome, SOFA=sequential organ failure assessment, INR=international normalized ratio

*, <0.00833 vs. 70–139 mg/dL, **, <0.00833 vs. 140–179 mg/dL, #, <0.00833 vs. >180 mg/dL
