# Supplementary material for: Impact of blood glucose abnormalities on outcomes and disease severity in patients with severe sepsis: An analysis from a multicenter, prospective survey of severe sepsis
Source: PLoS One. 2020 Mar 11;15(3):e0229919. doi: 10.1371/journal.pone.0229919 (PMC7065801; doi:10.1371/journal.pone.0229919)
Supplement: S2 Table — (DOCX) [file pone.0229919.s002.docx]

Supplementary Table 2. Clinical outcomes and blood glucose levels at admission in patients diagnosed with sepsis according to the Sepsis-3 criteria

| Outcomes | All patients  (n= 930) | Blood glucose <70 mg/dL  (n= 56) | Blood glucose 70–139 mg/dL  (n= 435) | Blood glucose 140–179 mg/dL  (n= 183) | Blood glucose >180 mg/dL  (n= 256) |  |
| --- | --- | --- | --- | --- | --- | --- |
| In-hospital mortality | 220/930, 23.7% | 21/56, 37.5% | 103/435, 23.7% | 37/183, 20.2% | 59/256, 23.0% | 0.066 |
| 28-day mortality | 175/930, 18.8% | 19/56, 33.9%*^,^ ** | 84/435, 19.3% | 31/183, 16.9% | 41/256, 16.0% | 0.004 |
| Survivor disposition at discharge | (n=710) | (n=35) | (n=332) | (n=146) | (n=197) | 0.094 |
| Home (n, %) | 243, 34.2% | 8, 22.9% | 117, 35.2% | 56, 38.3% | 62, 31.5% |  |
| Transfer (n, %) | 467, 65.8% | 27, 77.1% | 215, 64.8% | 90, 61.7% | 135, 68.5% |  |
| ICU-free days | 18 (9-23) | 16 (6-20.5) | 19 (11-24) | 17 (9-23) | 17 (8-23) | 0.123 |
| Ventilator-free days | 20 (0-27) | 14 (0-25) | 21 (0-28) | 19 (0-26) | 19 (0-25) | 0.079 |
| Length of hospital stay | 25 (13-47) | 27 (10.25-43.5) | 24 (12-45) | 27 (15-46) | 25 (15-51) | 0.327 |

Reported counts (proportions) for categorical variables and medians (interquartile ranges) for continuous variables.

Missing data: in-hospital mortality=29; 28-day mortality=39; ICU-free days=183; ventilator-free days=8; length of hospital stay=31

ICU=intensive care unit

*, p<0.00833 on comparison with the 140–179 mg/dL group; **, p<0.00833 on comparison with the >180 mg/dL group
