# Supplementary material for: Impact of blood glucose abnormalities on outcomes and disease severity in patients with severe sepsis: An analysis from a multicenter, prospective survey of severe sepsis
Source: PLoS One. 2020 Mar 11;15(3):e0229919. doi: 10.1371/journal.pone.0229919 (PMC7065801; doi:10.1371/journal.pone.0229919)
Supplement: S3 Table — (DOCX) [file pone.0229919.s003.docx]

Supplementary Table 3. Clinical outcomes according to the quartiles of patient’s blood glucose levels at admission

| Outcomes | All patients  (n= 1158) | Blood glucose <105 mg/dL  (n= 292) | Blood glucose 105–136 mg/dL  (n= 288) | Blood glucose 137–186 mg/dL  (n= 294) | Blood glucose >187 mg/dL  (n= 284) |  |
| --- | --- | --- | --- | --- | --- | --- |
| In-hospital mortality | 266/1127, 23.6% | 84/292, 28.8%* | 55/288, 19.1% | 61/294, 20.7% | 66/284, 23.2% | 0.030 |
| 28-day mortality | 213/1116, 19.1% | 70/292, 23.4% | 45/288, 15.6% | 51/294, 17.3% | 47/284, 16.5% | 0.035 |
| Survivor disposition at discharge | (n=861) | (n=203) | (n=228) | (n=220) | (n=210) | 0.014 |
| Home | 317, 36.8% | 64, 31.5% | 94, 41.2% | 92, 41.8% | 67, 31.9% |  |
| Transfer | 544, 63.2% | 139, 68.5% | 134, 58.8% | 128, 58.2% | 143, 68.1% |  |
| ICU-free days | 19 (11-24) | 20(10-24) | 20 (13-24) | 19 (11-24) | 18 (9-23) | 0.075 |
| Ventilator-free days | 21 (0-28) | 21 (0-28) | 22 (0.25-28) | 21 (0-28) | 19 (0-26) | 0.169 |
| Length of hospital stay | 24 (12-46) | 23 (10-45) | 23 (11-45) | 25 (14-40) | 23.5 (13.25-51) | 0.335 |

Reported counts (proportions) for categorical variables and medians (interquartile ranges) for continuous variables.

Missing data: in-hospital mortality=31; 28-day mortality=42; ICU-free days=255; ventilator-free days=42; length of hospital stay=31

ICU=intensive care unit

*, p<0.00833 on comparison with the 105-136 mg/dL group.
