# Supplementary material for: Impact of blood glucose abnormalities on outcomes and disease severity in patients with severe sepsis: An analysis from a multicenter, prospective survey of severe sepsis
Source: PLoS One. 2020 Mar 11;15(3):e0229919. doi: 10.1371/journal.pone.0229919 (PMC7065801; doi:10.1371/journal.pone.0229919)
Supplement: S4 Table — (DOCX) [file pone.0229919.s004.docx]

Supplementary Table 4. In-hospital mortality and body glucose category on admission

| Glucose categories | Mortality (n, %) | Unadjusted  odds ratio | 95% confidence interval | P value |
| --- | --- | --- | --- | --- |
| All patients |  |  |  |  |
| <70 mg/dL | 26/68, 38.2% | 2.06 | 1.22-3.49 | 0.006 |
| 70–139 mg/dL | 123/533, 23.1% | reference | | |
| 140–179 mg/dL | 43/221, 19.5% | 0.81 | 0.85-1.19 | 0.275 |
| >180 mg/dL | 74/305, 24.3% | 1.07 | 0.77-1.49 | 0.697 |
|  | | | | |
| Patients with known diabetes mellitus | |  |  |  |
| <70 mg/dL | 6/13, 46.2% | 1.71 | 0.54-5.51 | 0.527 |
| 70–139 mg/dL | 22/66, 33.3% | reference | | |
| 140–179 mg/dL | 12/42, 28.6% | 0.80 | 0.35-1.84 | 0.603 |
| >180 mg/dL | 32/140, 22.9% | 0.59 | 0.31-1.13 | 0.128 |
| Patients without known diabetes mellitus | | |  |  |
| <70 mg/dL | 20/55, 36.4% | 2.07 | 1.15-3.72 | 0.014 |
| 70–139 mg/dL | 101/467, 21.6% | reference | | |
| 140–179 mg/dL | 31/179, 17.3% | 0.76 | 0.49-1.18 | 0.224 |
| >180 mg/dL | 42/165, 25.5% | 1.24 | 0.82-1.87 | 0.313 |
|  | | | | |
| Patients with septic shock | | |  |  |
| <70 mg/dL | 22/56, 39.3% | 1.71 | 0.96-3.07 | 0.069 |
| 70–139 mg/dL | 94/343, 27.4% | reference | | |
| 140–179 mg/dL | 26/123, 21.1% | 0.71 | 0.44-1.16 | 0.173 |
| >180 mg/dL | 55/182, 30.2% | 1.15 | 0.77-1.70 | 0.496 |
| Patients without septic shock | | | | |
| <70 mg/dL | 4/12, 33.3% | 2.78 | 0.84-9.31 | 0.112 |
| 70–139 mg/dL | 29/190, 15.3% | reference | | |
| 140–179 mg/dL | 17/98, 17.3% | 1.17 | 0.61-2.23 | 0.647 |
| >180 mg/dL | 19/123, 15.4% | 1.01 | 0.54-1.89 | 0.965 |
