# Supplementary material for: Impact of blood glucose abnormalities on outcomes and disease severity in patients with severe sepsis: An analysis from a multicenter, prospective survey of severe sepsis
Source: PLoS One. 2020 Mar 11;15(3):e0229919. doi: 10.1371/journal.pone.0229919 (PMC7065801; doi:10.1371/journal.pone.0229919)
Supplement: S5 Table — (DOCX) [file pone.0229919.s005.docx]

Supplementary Table 5.

In-hospital mortality in patients with or without hypoglycemia (<70 mg/dL) and septic shock

|  | Mortality (n, %) | Unadjusted  odds ratio | 95% confidence interval | P value |
| --- | --- | --- | --- | --- |
| Glucose <70 mg/dL  with Septic shock | 22/56, 39.3% | 3.44 | 1.90-6.24 | <0.001 |
| Glucose <70 mg/dL  without Septic shock | 4/12, 33.3% | 2.66 | 0.83-8.58 | 0.115 |
| Glucose >70 mg/dL  with Septic shock | 175/648, 27.0% | 1.97 | 1.44-2.70 | <0.001 |
| Glucose >70 mg/dL  without Septic shock | 65/411, 15.8% | reference | | |
