# Supplementary material for: Impact of blood glucose abnormalities on outcomes and disease severity in patients with severe sepsis: An analysis from a multicenter, prospective survey of severe sepsis
Source: PLoS One. 2020 Mar 11;15(3):e0229919. doi: 10.1371/journal.pone.0229919 (PMC7065801; doi:10.1371/journal.pone.0229919)
Supplement: S6 Table — (DOCX) [file pone.0229919.s006.docx]

**FORECAST Study Group**

List of FORECAST Study Group and all of the institutional ethics committees reviewed and approved this study

1. Nagasaki University Hospital (Osamu Tasaki)
2. Osaka City University Hospital (Yasumitsu Mizobata)
3. Tokyobay Urayasu Ichikawa Medical Center (Hiraku Funakoshi)
4. Aso Iizuka Hospital (Toshiro Okuyama)
5. Tomei Atsugi Hospital (Iwao Yamashita)
6. Hiratsuka City Hospital (Toshio Kanai)
7. National Hospital Organization Sendai Medical Center (Yasuo Yamada)
8. Ehime University Hospital (Mayuki Aibiki)
9. Okayama University Hospital (Keiji Sato)
10. Tokuyama Central Hospital (Susumu Yamashita)
11. Fukuyama City Hospital (Susumu Yamashita)
12. JA Hiroshima General Hospital (Kenichi Yoshida)
13. Kumamoto University Hospital (Shunji Kasaoka)
14. Hachinohe City Hospital (Akihide Kon)
15. Osaka City General Hospital (Hiroshi Rinka)
16. National Hospital Organization Disaster Medical Center (Hiroshi Kato)
17. University of Toyama (Hiroshi Okudera)
18. Sapporo Medical University (Eichi Narimatsu)
19. Okayama Saiseikai General Hospital (Toshifumi Fujiwara)
20. Juntendo University Nerima Hospital (Manabu Sugita)
21. National Hospital Organization Hokkaido Medical Center (Yasuo Shichinohe)
22. Akita University Hospital (Hajime Nakae)
23. Japanese Red Cross Society Kyoto Daini Hospital (Ryouji Iiduka)
24. Maebashi Red Cross Hospital (Mitsunobu Nakamura)
25. Sendai City Hospital (Yuji Murata)
26. Subaru Health Insurance Society Ota Memorial Hospital (Yoshitake Sato)
27. Fukuoka University Hospital (Hiroyasu Ishikura)
28. Ishikawa Prefectural Central Hospital (Yasuhiro Myojo)
29. Shiga University of Medical Science (Yasuyuki Tsujita)
30. Nihon University School of Medicine (Kosaku Kinoshita)
31. Seirei Yokohama General Hospital (Hiroyuki Yamaguchi)
32. National Hospital Organization Kumamoto Medical Center (Toshihiro Sakurai)
33. Saiseikai Utsunomiya Hospital (Satoru Miyatake)
34. National Hospital Organization Higashi-Ohmi General Medical Center (Takao Saotome)
35. National Hospital Organization Mito Medical Center (Susumu Yasuda)
36. Tsukuba Medical Center Hospital (Toshikazu Abe)
37. Osaka University Graduate School of Medicine (Hiroshi Ogura, Yutaka Umemura)
38. Kameda Medical Center (Atsushi Shiraishi)
39. Tohoku University Graduate School of Medicine (Shigeki Kushimoto)
40. National Defense Medical College (Daizoh Saitoh)
41. Keio University School of Medicine (Seitaro Fujishima, Junichi Sasaki)
42. University of Occupational and Environmental Health (Toshihiko Mayumi)
43. Kawasaki Medical School (Yasukazu Shiino)
44. Chiba University Graduate School of Medicine (Taka-aki Nakada)
45. Kyorin University School of Medicine (Takehiko Tarui)
46. Kagawa University Hospital (Toru Hifumi)
47. Tokyo Medical and Dental University (Yasuhiro Otomo)
48. Hyogo College of Medicine (Joji Kotani)
49. Saga University Hospital (Yuichiro Sakamoto)
50. Aizu Chuo Hospital (Shin-ichiro Shiraishi)
51. Kawasaki Municipal Kawasaki Hospital (Kiyotsugu Takuma)
52. Yamaguchi University Hospital (Ryosuke Tsuruta)
53. Center Hospital of the National Center for Global Health and Medicine (Akiyoshi Hagiwara)
54. Osaka General Medical Center (Kazuma Yamakawa)
55. Aichi Medical University Hospital (Naoshi Takeyama)
56. Kurume University Hospital (Norio Yamashita)
57. Teikyo University School of Medicine (Hiroto Ikeda)
58. Rinku General Medical Center (Yasuaki Mizushima)
59. Hokkaido University Graduate School of Medicine (Satoshi Gando)
