# Supplementary material for: Impact of blood glucose abnormalities on outcomes and disease severity in patients with severe sepsis: An analysis from a multicenter, prospective survey of severe sepsis
Source: PLoS One. 2020 Mar 11;15(3):e0229919. doi: 10.1371/journal.pone.0229919 (PMC7065801; doi:10.1371/journal.pone.0229919)
Supplement: Attachment — Submitted filename: Plos One dysglycemia.docx [file pone.0229919.s008.docx]

Summary

Thank you for the opportunity to review this paper. This is a very interesting study that aims to elucidate the predictive role of hypoglycemia in septic patients. The authors utilize a large prospective cohort study to answer the question of whether initial hypoglycemia is predictive of outcome. The results are interesting, but I have issues with how the data was analyzed and presented. Hepatic failure needs to be accounted for specifically in the analyses. As it is currently presented, I think this study is at risk of making the assumption that hypoglycemia is predictive of death, when it is actually just associated with severe disease.

Major points

The study objective to elucidate dysglycemia in a sepsis population is clinically relevant

The authors state “initial blood glucose” level but do not provide timing of this initial glucose or how this was obtained. Was this from finger stick measurements, blood chemistries, etc? Also what was the mean time to measurement of this value from enrollment into the study? It is important to know where in the course of their disease the hypoglycemia occurred.

Even though the parent study (FORECAST) was designed prior to Sepsis-3, they should still report the proportion of patients meeting Sepsis-3 definitions. This should not be difficult given that the authors have SOFA score data available and will make the study more directly comparable to more recent studies.

The authors provide data to support for their choice of blood glucose groups. It would be important to know what they learned from their own data, rather than to utilize prior categorizations of blood glucose from other work. Separating the blood glucose measurements of their patients by quartiles (or similar) and looking at outcomes based on this would be informative.

How did the authors handle censored data for SOFA score in patients who died early?

A larger proportion of patients in the hypoglycemia group had glucose mod to severe liver disease. It is not surprising that those patients are more prone to hypoglycemia. Also, the SOFA scores of the hypoglycemia patients are significantly higher. It is important to know what the Hepatic SOFA scores are for the patients with hypoglycemia. The predictive ability of hypoglycemia could be confounded by liver disease or acute liver failure.

Given the potential interaction between liver disease/failure above it is important to add lactate levels (initial and repeat) to Table 1 and compare across groups.

Corticosteroid requirement is much higher in hypoglycemia group. Is there any data on whether the glucose levels were obtained prior to administration of corticosteroids or if these are post-steroid measures?

The presentation of the data is confusing. The authors mention Kaplay-Meier curves for survival, but then use a Cox regression. But they also report unadjusted odds ratios. If the outcome of interest is the association with mortality, I don’t think a Cox regression is needed as the time to death is not really that relevant. I also do not think unadjusted odds ratios should be presented, and should only be added as supplementary material. The Cox regression shows a higher hazard ratio for glucose < 70, which was robust to sensitivity analysis I think components of SOFA, specifically liver failure should be evaluated in the model as well as Charlson score for mod to severe liver disease. I think we are at risk of finding that hypoglycemia is associated with severely ill patients at risk of mortality, rather than predictive of itself.

Why are unadjusted odds ratios displayed in Table 3 and 4, rather than adjusted? These should not be displayed though they can be presented in the body of the manuscript, briefly.

Minor

Would prefer to see the tables separately and not in the body of the text, which makes it harder to read

Also the authors should indicate units appropriately in tables (N, %) in the first column

Odds ratios should only be reported to 1 or 2 places after the decimal
