# Supplementary material for: Impact of blood glucose abnormalities on outcomes and disease severity in patients with severe sepsis: An analysis from a multicenter, prospective survey of severe sepsis
Source: PLoS One. 2020 Mar 11;15(3):e0229919. doi: 10.1371/journal.pone.0229919 (PMC7065801; doi:10.1371/journal.pone.0229919)
Supplement: Attachment — Submitted filename: 20MQVO284779_Plos One dysglycemia_200102.docx [file pone.0229919.s009.docx]

Reviewer #1:

Summary

Thank you for the opportunity to review this paper. This is a very interesting study that aims to elucidate the predictive role of hypoglycemia in septic patients. The authors utilize a large prospective cohort study to answer the question of whether initial hypoglycemia is predictive of outcome. The results are interesting, but I have issues with how the data was analyzed and presented. Hepatic failure needs to be accounted for specifically in the analyses. As it is currently presented, I think this study is at risk of making the assumption that hypoglycemia is predictive of death, when it is actually just associated with severe disease.

(Response)

We thank the Reviewer for the favorable comments and acknowledgment of our work. Accordingly, we have revised our manuscript. To facilitate the review process, the revised sentences are shown in red in the revised manuscript.

Major points

The study objective to elucidate dysglycemia in a sepsis population is clinically relevant

The authors state “initial blood glucose” level but do not provide timing of this initial glucose or how this was obtained. Was this from finger stick measurements, blood chemistries, etc? Also what was the mean time to measurement of this value from enrollment into the study? It is important to know where in the course of their disease the hypoglycemia occurred.

(Response)

Thank you for your comments. Accordingly, we have added the following information in the methods and limitations section:

Methods: Blood glucose levels were measured using a blood gas analyzer (and not a glucometer) as recommend by Surviving Sepsis Campaign: International Guidelines for Management of Severe Sepsis and Septic Shock: 2012 (14). All glucose levels were measured prior to the administration of corticosteroids (if required).

Limitations: Third, although we used the blood glucose levels measured during initial evaluation and management, the time between measurement and the diagnosis of severe sepsis or septic shock was not defined in this registry. These measurement related issues may have influenced the results.

Even though the parent study (FORECAST) was designed prior to Sepsis-3, they should still report the proportion of patients meeting Sepsis-3 definitions. This should not be difficult given that the authors have SOFA score data available and will make the study more directly comparable to more recent studies.

(Response)

Thank you for your comments. We have added the outcome data for patients diagnosed with sepsis by the Sepsis-3 definition in Supplementary Table 2. For defining an acute increase in SOFA scores of ≥2, a baseline SOFA score of 0 was assumed in patients without a diagnosis of any chronic disease, as defined per APACHE II scores. If a chronic disease was present, a baseline SOFA score of 2 was assigned, similar to methods used in a previous study (JAMA. 2016;317:290–300).

Supplementary Table 2. Clinical outcomes and blood glucose levels at admission in patients diagnosed with sepsis according to the Sepsis-3 criteria

| Outcomes | All patients  (n= 930) | Blood glucose <70 mg/dL  (n= 56) | Blood glucose 70–139 mg/dL  (n= 435) | Blood glucose 140–179 mg/dL  (n= 183) | Blood glucose ≥180 mg/dL  (n= 256) |  |
| --- | --- | --- | --- | --- | --- | --- |
| In-hospital mortality | 220/930, 23.7% | 21/56, 37.5% | 103/435, 23.7% | 37/183, 20.2% | 59/256, 23.0% | 0.066 |
| 28-day mortality | 175/930, 18.8% | 19/56, 33.9%*^,^ ** | 84/435, 19.3% | 31/183, 16.9% | 41/256, 16.0% | 0.004 |
| Survivor disposition at discharge | (n=710) | (n=35) | (n=332) | (n=146) | (n=197) | 0.094 |
| Home (n, %) | 243, 34.2% | 8, 22.9% | 117, 35.2% | 56, 38.3% | 62, 31.5% |  |
| Transfer (n, %) | 467, 65.8% | 27, 77.1% | 215, 64.8% | 90, 61.7% | 135, 68.5% |  |
| ICU-free days | 18 (9-23) | 16 (6-20.5) | 19 (11-24) | 17 (9-23) | 17 (8-23) | 0.123 |
| Ventilator-free days | 20 (0-27) | 14 (0-25) | 21 (0-28) | 19 (0-26) | 19 (0-25) | 0.079 |
| Length of hospital stay | 25 (13-47) | 27 (10.25-43.5) | 24 (12-45) | 27 (15-46) | 25 (15-51) | 0.327 |

Reported counts (proportions) for categorical variables and medians (interquartile ranges) for continuous variables.

Missing data: in-hospital mortality=29; 28-day mortality=39; ICU-free days=183; ventilator-free days=8; length of hospital stay=31

ICU=intensive care unit

*, p<0.00833 on comparison with the 140–179 mg/dL group; **, p<0.00833 on comparison with the ≥180 mg/dL group

The authors provide data to support for their choice of blood glucose groups. It would be important to know what they learned from their own data, rather than to utilize prior categorizations of blood glucose from other work. Separating the blood glucose measurements of their patients by quartiles (or similar) and looking at outcomes based on this would be informative.

(Response)

Thank you for your comments. Although we performed our analysis based on pre-defined categorization of blood glucose levels to recognize the importance of hypoglycemia (70 mg/dL or less), it is important to know the findings obtained from our own data. As such, we have presented all outcomes according to quartiles of patient glucose levels in Supplementary Table 3.

Supplementary Table 3. Clinical outcomes according to the quartiles of patient’s blood glucose levels at admission

| Outcomes | All patients  (n= 1158) | Blood glucose <105 mg/dL  (n= 292) | Blood glucose 105–136 mg/dL  (n= 288) | Blood glucose 137–186 mg/dL  (n= 294) | Blood glucose ≥187 mg/dL  (n= 284) |  |
| --- | --- | --- | --- | --- | --- | --- |
| In-hospital mortality | 266/1127, 23.6% | 84/292, 28.8%* | 55/288, 19.1% | 61/294, 20.7% | 66/284, 23.2% | 0.030 |
| 28-day mortality | 213/1116, 19.1% | 70/292, 23.4% | 45/288, 15.6% | 51/294, 17.3% | 47/284, 16.5% | 0.035 |
| Survivor disposition at discharge | (n=861) | (n=203) | (n=228) | (n=220) | (n=210) | 0.014 |
| Home | 317, 36.8% | 64, 31.5% | 94, 41.2% | 92, 41.8% | 67, 31.9% |  |
| Transfer | 544, 63.2% | 139, 68.5% | 134, 58.8% | 128, 58.2% | 143, 68.1% |  |
| ICU-free days | 19 (11-24) | 20(10-24) | 20 (13-24) | 19 (11-24) | 18 (9-23) | 0.075 |
| Ventilator-free days | 21 (0-28) | 21 (0-28) | 22 (0.25-28) | 21 (0-28) | 19 (0-26) | 0.169 |
| Length of hospital stay | 24 (12-46) | 23 (10-45) | 23 (11-45) | 25 (14-40) | 23.5 (13.25-51) | 0.335 |

Reported counts (proportions) for categorical variables and medians (interquartile ranges) for continuous variables.

Missing data: in-hospital mortality=31; 28-day mortality=42; ICU-free days=255; ventilator-free days=42; length of hospital stay=31

ICU=intensive care unit

*, p<0.00833 on comparison with the 105-136 mg/dL group.

How did the authors handle censored data for SOFA score in patients who died early?

(Response)

Thank you for your comments. The SOFA score was calculated using physiological and laboratory values determined during the initial evaluation. Therefore, we did not handle censored data for SOFA scores.

We have added the following information in the methods section:

The Sequential Organ Failure Assessment (SOFA) score was calculated using physiological and laboratory values during the initial evaluation.

A larger proportion of patients in the hypoglycemia group had glucose mod to severe liver disease. It is not surprising that those patients are more prone to hypoglycemia. Also, the SOFA scores of the hypoglycemia patients are significantly higher. It is important to know what the Hepatic SOFA scores are for the patients with hypoglycemia. The predictive ability of hypoglycemia could be confounded by liver disease or acute liver failure.

Given the potential interaction between liver disease/failure above it is important to add lactate levels (initial and repeat) to Table 1 and compare across groups.

(Response)

Thank you for your comments. We have added lactate levels in Table 1 and the hepatic SOFA scores in the main text according to the reviewer’s comments. However, we have no repeated values of lactate levels in this dataset.

Added data and information:

Lactate levels in Table 1: 3.00 (1.80-5.30), 5.50 (3.05- 9.75), 2.70 (1.60- 5.10), 2.80 (1.78- 4.93), 3.25 (2.18-5.60), respectively.

Hepatic SOFA scores in the main text (Results section): The hepatic SOFA scores in the categories with glucose levels of <70, 70-139, 140-179, and >180 mg/dL were 1 (0-1), 0 (0-1), 0 (0-1), and 0 (0-1), respectively (p<0.001).

We have also added the following comments:

This score was significantly higher in the group with patients with blood glucose levels <70 mg/dL than in the other groups.

Corticosteroid requirement is much higher in hypoglycemia group. Is there any data on whether the glucose levels were obtained prior to administration of corticosteroids or if these are post-steroid measures?

(Response)

Thank you for your comments. We have added the following information in the methods section:

All glucose levels were measured prior to the administration of corticosteroids (if required).

The authors mention Kaplan-Meier curves for survival, but then use a Cox regression. But they also report unadjusted odds ratios. If the outcome of interest is the association with mortality, I don’t think a Cox regression is needed as the time to death is not really that relevant. I also do not think unadjusted odds ratios should be presented, and should only be added as supplementary material. The Cox regression shows a higher hazard ratio for glucose < 70, which was robust to sensitivity analysis I think components of SOFA, specifically liver failure should be evaluated in the model as well as Charlson score for mod to severe liver disease. I think we are at risk of finding that hypoglycemia is associated with severely ill patients at risk of mortality, rather than predictive of itself.

Why are unadjusted odds ratios displayed in Table 3 and 4, rather than adjusted? These should not be displayed though they can be presented in the body of the manuscript, briefly.

(Response)

Thank you for your comments. The Kaplan-Meier curves for survival were deleted from the main body of the manuscript and presented as supplementary content. Tables 3 and 4 are presented without the unadjusted odds ratios in the main body of the manuscript, and odds ratios are presented in Supplementary Tables 4 and 5.

The reviewer has highlighted the significance of the coexistence of moderate-to-severe liver disease. Although these comments were reasonable, coexisting moderate-to-severe liver disease was only present in 26 patients showing significantly higher mortality and prevalence of hypoglycemia (Mortality: 255/1132, 22.5% vs. 11/26, 42.3%, p=0.011; Hypoglycemia: 61/1132, 5.4% vs 8/26, 30.8%, p<0.001). Based on these interactions, we did not evaluate the coexistence of moderate-to-severe liver disease as an explanatory variable in this study.

In addition, the outcome of interest was not only mortality but also the duration of survival, as demonstrated in the Kaplan-Meier survival curves. Therefore, we evaluated the significance of hypoglycemia using Cox regression analysis.

Minor

Would prefer to see the tables separately and not in the body of the text, which makes it harder to read

(Response)

We have revised the manuscript as per your suggestion.

Also the authors should indicate units appropriately in tables (N, %) in the first column

(Response)

We add the (N, %) in the tables as per your suggestion.

Odds ratios should only be reported to 1 or 2 places after the decimal

(Response)

We have revised the description of odds ratios and hazard ratios as per your suggestion.

Reviewer #2: An interesting subgroup analysis highlighting the association between hypoglycemia on admission and mortality in severe sepsis patients from a previously performed multicenter prospective cohort. Highlighting an association between hypoglycemia in non-diabetic sepsis patients and increased mortality is a relevant finding.

Appropriate statistics used for the most part and findings correctly described as an association only in need of further study to determine causation.

(Response)

We thank the Reviewer for the favorable comments and acknowledgment of our work. Accordingly, we have revised our manuscript. To facilitate the review process, the revised sentences are shown in red in the revised manuscript.

The fact that the cohort is a mixed ICU population including medical and trauma patients may confound some of the data a bit. Would be interesting to see if the association still stands in both the trauma and medical ICU subgroups or if it would change.

(Response)

Thank you for your comments. We agree with the Reviewer’s comments. However, the dataset of the FORECAST Sepsis cohort has no information on trauma and medical ICU subgroups.

Abstract still has a few errors and needs some rewording to aid in understanding for readers.

(Response)

Thank you for your comments. We revised the abstract as you kindly suggested. In addition, we revised some sentences according to the changes made in the main manuscript.
