# Supplementary material for: Impact of blood glucose abnormalities on outcomes and disease severity in patients with severe sepsis: An analysis from a multicenter, prospective survey of severe sepsis
Source: PLoS One. 2020 Mar 11;15(3):e0229919. doi: 10.1371/journal.pone.0229919 (PMC7065801; doi:10.1371/journal.pone.0229919)
Supplement: Attachment — Submitted filename: PONE-D-19-25111R2.200213.docx [file pone.0229919.s010.docx]

Editor’s comments

Thank you for your time and effort revising the manuscript, which has been substantially improved. I am in agreement with the reviewer about the potential confounding effect of liver disease--are the poor outcomes related to hypoglycemia itself, or is this simply a manifestation of liver disease? With only 26 patients in this category, I think it would be difficult to make any firm conclusions about which factor drove the outcome (ie, an analysis comparing liver disease with vs without hypoglycemia would be under-powered). However, I do think it is worth addressing, most substantively in the discussion, as well mentioning this in the abstract.

(Response)

We thank the Editor for the favorable comments and acknowledgment of our work. Accordingly, we have revised our manuscript. To facilitate the review process, the revised sentences are shown in red in the revised manuscript. According to the Editor’s comments, we revised the manuscript as follows.

We have added the following comments to the discussion section:

The liver plays an important role in glucose metabolism, and hypoglycemia may occur due to impaired ability of the liver to increase plasma glucose through gluconeogenesis (31). Hypoglycemia has also been reported as a common manifestation of sepsis patients in cirrhosis (32). Although the hepatic SOFA score in hypoglycemic patients was significantly higher than those in other groups, the presence of "moderate to severe liver disease" as a comorbidity was not different between hypoglycemia and non-hypoglycemia patients in this study. In addition, hepatic SOFA score on admission in survivors was not higher than that in non-survivors, and the odds ratios of hepatic SOFA scores of ≥1 for in-hospital mortality and 28-day mortality were 1.18 (0.89–1.57) and 1.22 (0.90–1.66), respectively. Hypoglycemia may be related to hepatic impairment accompanied by corticosteroid insufficiency, and further study including a larger number of patients is required to elucidate the impact of the relationship between hypoglycemia and hepatic impairment on the outcome of patients with sepsis.

We have also added the following comments in results section of the abstract:

Of 1158 patients, 69, 543, 233, and 313 patients were categorized as glucose levels <70 (hypoglycemia), 70-139, 140-179, >180 mg/dL, respectively. Both the Acute Physiological and Chronic Health Evaluation II and Sequential Organ Failure Assessment (SOFA) scores on the day of enrollment were higher in the hypoglycemia patients than non-hypoglycemic patients than in those with 70-179 mg/dL. The hepatic SOFA scores were also higher in hypoglycemia patients.

Can you also include in the methods what criteria were used to classify patients as "moderate to severe liver disease"? I think this would also be informative to readers.

(Response)

We have provided the following definitions in the methods section: Severe, moderate, and mild liver disease as comorbidities were defined as follows: severe; cirrhosis and portal hypertension with variceal bleeding history; moderate, cirrhosis and portal hypertension but no variceal bleeding history; and mild, chronic hepatitis (or cirrhosis without portal hypertension) (23).

Also, I think the first line of the abstract should read "Dysglycemia **is**..." not "Dysglycemia **are**..."

➠ We have revised as per your suggestion.

Reviewer #1:

Overall the authors have addressed most of my concerns.

However, now that they have identified the significant influence of hepatic disease (elevated hepatic SOFA) in the subgroup with glucose < 70, the reader needs to be sure that hepatic failure isn't driving most of the difference seen in this group. I am not sure based on reviewing their regression models that liver failure was fully accounted for here. It is not explicitly stated nor made clear in the methods. This point may require a statistical review.

(Response)

We thank the Reviewer for the favorable comments and acknowledgment of our work. Accordingly, we have revised our manuscript. To facilitate the review process, the revised sentences are shown in red in the revised manuscript.

We have added the following comments in the methods and results sections:

Methods:

**Hepatic disease/dysfunction, glycemic abnormalities, Sepsis-3, and outcomes**

To evaluate the relationship between hepatic impairment and glycemic abnormalities, the presence of moderate to severe hepatic disease as a comorbidity and hepatic SOFA score were compared among groups. In addition, to assess the influence of hepatic impairment on the outcome of hypoglycemia, the presence of moderate to severe hepatic disease and hepatic SOFA score >0 were compared between hypoglycemia and non-hypoglycemia patients. Severe, moderate, and mild liver disease as comorbidities were defined as follows: severe; cirrhosis and portal hypertension with variceal bleeding history; moderate, cirrhosis and portal hypertension but no variceal bleeding history; and mild, chronic hepatitis (or cirrhosis without portal hypertension).

Results:

Influence of hepatic disease/dysfunction on mortality

The hepatic SOFA scores in the patients who were categorized according to their glucose levels of <70, 70-139, 140-179, and >180 mg/dL were 1 (0-1), 0 (0-1), 0 (0-1), and 0 (0-1), respectively (p<0.001). The score was significantly higher in the blood glucose level <70 mg/dL group than in the other groups (Table 1).

In contrast, the presence of "moderate to severe liver disease" as a comorbidity was not different between hypoglycemic and non-hypoglycemic patients (8/69, 151/1089, p =0.719). Also, hepatic SOFA on admission in survivors was not higher than that of non-survivors (p=0.232). Odds ratios of hepatic SOFA score of 1 or more for in-hospital mortality and 28-day mortality were 1.18 (0.89–1.57) and 1.22 (0.90–1.66), respectively.

Moreover, I believe the finding of significantly elevated hepatic SOFA should be mentioned in the abstract, discussion and conclusion.

(Response)

We have added the following comments in the abstract, discussion, and conclusion sections of the revised manuscript:

Results section of the abstract:

Of the 1158 enrolled patients, 69, 543, 233, and 313 patients were categorized according to their glucose levels, i.e., <70 (hypoglycemia), 70-139, 140-179, >180 mg/dL, respectively. Both the Acute Physiological and Chronic Health Evaluation II and Sequential Organ Failure Assessment (SOFA) scores on the day of enrollment were higher in the hypoglycemia patients than non-hypoglycemic patients than in those with 70-179 mg/dL. The hepatic SOFA scores were also higher in hypoglycemia patients.

Discussion:

The liver plays an important role in glucose metabolism, and hypoglycemia may occur due to impaired ability of the liver to increase plasma glucose through gluconeogenesis (31). Hypoglycemia has also been reported as a common manifestation of sepsis patients in cirrhosis (32). Although the hepatic SOFA score in hypoglycemic patients was significantly higher than those in other groups, the presence of "moderate to severe liver disease" as a comorbidity was not different between hypoglycemia and non-hypoglycemia patients in this study. In addition, hepatic SOFA score on admission in survivors was not higher than that in non-survivors, and the odds ratios of hepatic SOFA scores of ≥1 for in-hospital mortality and 28-day mortality were 1.18 (0.89–1.57) and 1.22 (0.90–1.66), respectively. Hypoglycemia may be related to hepatic impairment accompanied by corticosteroid insufficiency, and further study including a larger number of patients is required to elucidate the impact of the relationship between hypoglycemia and hepatic impairment on the outcome of patients with sepsis.

Conclusions

Hypoglycemia may be related to increased disease severity and a higher mortality rate in patients with sepsis, and moreover, was an independent predictor of poor clinical outcome. These relationships were evident only in patients without diabetes. Patients with both hypoglycemia and septic shock had increase mortality rates. Although hypoglycemia may be related to hepatic impairment, further studies are needed to explicate the relationship with respect to the outcomes of patients with sepsis.
